# Reduced α-galactosidase A activity in zebrafish (*Danio rerio)* mirrors distinct features of Fabry nephropathy phenotype

**DOI:** 10.1016/j.ymgmr.2022.100851

**Published:** 2022-02-17

**Authors:** Hassan O.A. Elsaid, Jessica Furriol, Maria Blomqvist, Mette Diswall, Sabine Leh, Naouel Gharbi, Jan Haug Anonsen, Janka Babickova, Camilla Tøndel, Einar Svarstad, Hans-Peter Marti, Maximilian Krause

**Affiliations:** a Department of Clinical Medicine, University of Bergen, Bergen, Norway; b Department of Medicine, Haukeland University Hospital, Bergen, Norway; c Department of Clinical Chemistry, Sahlgrenska University Hospital, Gothenburg, Sweden; d Department of Laboratory Medicine, Institute of Biomedicine, Sahlgrenska Academy, University of Gothenburg, Gothenburg, Sweden; e Department of Pathology, Haukeland University Hospital, Bergen, Norway; f Department of Climate & Environment, Industrial Biotechnology, NORCE, Bergen, Mekjarvik, Norway; g Institute of Molecular Biomedicine, Faculty of Medicine, Comenius University, Bratislava, Slovakia; h Department of Pediatrics, Haukeland University Hospital, Bergen, Norway; i Computational Biology Unit, Department of Informatics, University of Bergen, Bergen, Norway; j Sars Centre for Molecular Marine Biology, University of Bergen, Bergen, Norway

**Keywords:** *GLA*, α-Galactosidase A, α-GAL activity, Zebrafish, Fabry disease

## Abstract

Fabry disease (FD) is a rare genetic lysosomal storage disorder, resulting from partial or complete lack of alpha-galactosidase A (α-GAL) enzyme, leading to systemic accumulation of substrate glycosphingolipids with a broad range of tissue damage. Current *in vivo* models are laborious, expensive, and fail to adequately mirror the complex FD physiopathology. To address these issues, we developed an innovative FD model in zebrafish.

Zebrafish *GLA* gene encoding α-GAL enzyme presents a high (>70%) homology with its human counterpart, and the corresponding protein has a similar tissue distribution, as evaluated by immunohistochemistry. Moreover, a similar enzymatic activity in different life stages could be demonstrated. By using CRISPR/Cas9 technology, we generated a mutant zebrafish with decreased *GLA* gene expression, and decreased expression of the specific gene product in the kidney. Mutant animals showed higher plasma creatinine levels and proteinuria. Transmission electron microscopy (TEM) studies documented an increased podocyte foot process width (FPW) in mutant, as compared to wild type zebrafish.

This zebrafish model reliably mirrors distinct features of human FD and could be advantageously used for the identification of novel biomarkers and for an effective screening of innovative therapeutic approaches.

## Highlights

• Zebrafish's galactosidase, alpha gene (*GLA)* is the homologous to human gene.
• Zebrafish α-GAL protein expressed in kidneys, similarly to humans, is functionally active.
• Fishes with mutated *GLA* have high plasma creatinine and proteinuria.
• *GLA*-mutant Zebrafish reliably models human Fabry disease.

## 1 Introduction

Fabry disease (FD) is a rare X-linked lysosomal storage disorder caused by a variety of mutations in the alpha-galactosidase gene (*GLA)* on *Xq21.3-q*22. The result of which is a wide spectrum of α-GAL enzyme activities, ranging from normal to complete deficiency [1], leading to different clinical phenotypes with both intra- and interfamilial clinical variabilities [2], [3].

As α-GAL hydrolyzes glycosphingolipids and glycopeptides [4], its complete deficiency leads to multi-organ accumulation of the glycosphingolipid globotriaosylceramide (Gb3) [5], but mainly in the kidney, heart, and nervous system [6], [7]. In the human kidney, the main clinical manifestations are due to Gb3 accumulation throughout the nephron and, predominantly, in renal epithelial cells [6]. Progressive Gb3 accumulation is associated with end-stage renal disease (ESRD) [8], [9].

Although Gb3 and the deacetylated form lysoGb3 in plasma are currently used for FD diagnosis and to monitor treatment effectiveness [10], [11], their ability to mirror all *GLA* gene mutations detected so far is debated [12], [13], [14], [15], [16].

Enzyme replacement therapy (ERT) has improved multiple aspects of FD. However, the associated complications *e.g.* allergies and variable efficiency that depend on the age and degree of nephropathy [17] as well as the elevated mortality rates, contribute to considering ERT as a disease modifier rather than a cure [18]. Therefore, innovative new treatment options are still urgently needed. Nevertheless, the development of novel therapies for FD critically requires the availability of adequate experimental models [17], [18], [19], [20].

*In vitro* studies using different cell lines may model the main FD hallmark, *e.g.* Gb3 accumulation [21], [22], [23], [24], but fail to address the complexity of damaged organs [25]. Instead, murine FD models [26], [27] recognized the role of ERT in decreasing Gb3 levels [28], and some of them succeeded in imitating the renal impairment of FD, however, hypertrophic cardiomyopathy was not reported in this FD model [29]. Fortunately, a study using FD model in rat have managed to recapitulate both renal and heart phenotypes [30], in addition, FD-related ocular manifestations was reported by the same research group [31]. However, knockout Fabry mice maintain a standard adult lifetime and their premature death was never attributed to the FD complication *i.e.* kidney or heart defects [29]. Others FD model appear clinically normal, and do not show obvious microscopic lesions in the kidney, liver, heart, spleen, lungs, and brain [32]. Therefore, current *in vitro* and *in vivo* models fail to fully elucidate FD physio-histopathology, and do not allow ERT optimization for personalized treatment. On top of that, murine studies can be laborious, and expensive, and it is challenging to follow the possible lack or reduction of α-GAL enzymatic activity early during intra-uterine gestation [25], [32].

Thus, additional *in vivo* Fabry disease models are needed to explore glomerular filtration barrier integrity and to facilitate screening for potential drugs [25], [32]. Moreover, inconsistency of Gb3 and lysoGb3 monitoring in kidney during treatment requires more out of the box thinking, including considering an animal model that is voided of Gb3/lysoGb3.

In this context, zebrafish is a good candidate since it lacks Gb3 synthase and, therefore, cannot produce Gb3 [33]. This could allow to investigate the substrate-independent role of *GLA* mutation and other low scale yet powerful markers that might be masked by the over-consideration of Gb3 and lysoGb3. Based on the unmistakable similarity of its kidney with the human one, zebrafish has extensively been used to study abnormalities in kidney development, inherited glomerulopathies, and ciliopathy-associated human cystic kidney diseases [34]. In the recent years, the use of zebrafish to study lysosomal storage disorders has grown tremendously [35], and with new advances in gene-editing technologies, researchers were able to produce zebrafish transgenic lines that facilitate the study of lysosomal storage disorders [36].

In addition to the similarities with human kidneys, zebrafish is valuable for other research-friendly characteristics such as ex-utero fertilization and development, rapid development from embryos to larvae in 5 days, and to full adult stage in 90 days, and high fecundity. Additionally, the transparency of embryos and larvae allow easy visualization of developmental processes or monitoring of the desired phenotype, [37]. Advantageously, the robust development of the pronephrone which is fully functioning at 4-day post-fertilization (dpf) identify zebrafish as an unavoidable organism in high throughput drug screening studies [34], [38], [39] and a useful bridge between *in vitro* cell- and *in vivo* rodent-based FD models [40].

On the basis of these considerations, we investigated the characteristics of α-GAL in wild-type zebrafish and evaluated the effects of the inhibition of *GLA* gene expression, to explore the possibility of using this valuable organism in FD studies.

## 2 Materials and methods

### 2.1 Ethical approval

The Norwegian Food Safety Authority (Mattilsynet) granted ethical approval for this study (FOTS ID 15256). All procedures were performed following standard protocols of the Zebrafish Facility, University of Bergen (UiB). We used the AB/Tübingen (AB/TU) strain of zebrafish for our experiments.

### 2.2 Zebrafish maintenance and sample collection

Eggs, embryos, larvae, juveniles, and adult fish were handled in compliance with applicable national and international standards, according to zebrafish facility regulation at the University of Bergen. Under normal laboratory conditions, an adult (90+ days post-fertilization dpf) wild-type zebrafish was held at 28 °C on a 14 h light/10 h dark period. Standard spawning protocol (www.zfin.org) was followed by egg harvesting. Eggs were stored in an E3 medium containing 0.01% methylene blue after harvesting. Embryos and larvae were incubated at 28 °C until 5 dpf. Current rules do not require permission for testing on zebrafish embryos before the free-feeding stage (5 dpf). According to the zebrafish facility rules, all invasive pain-causing interventions on stages older than 5 dpf were performed under anesthetic conditions.

### 2.3 Bioinformatics analysis and prediction of α-GAL structure in zebrafish

Using the BLAST algorithm with default parameters, we compared nucleotide and amino acid sequences to the non-redundant gene databases accessible at the National Centre for Biotechnology Information GenBank database (NCBI) http://blast.ncbi.nlm.nih.gov. NCBI BLAST (https://blast.ncbi.nlm.nih.gov/Blast.cgi) was used to evaluate sequence similarities against the Zebrafish May.2017 (GRCz11/danRer11) genome assembly. ClustalW was used to generate the multiple sequence alignment, which was then run on the Geneious software. The Ensembl db (http://www.ensembl.org) was used for comparative genomics analysis of *GLA* sequences in zebrafish and humans. The I-TASSER online modeling service was used to perform the homology modeling analysis [41]. The model was selected based on the confidence score (C-score), assessing the predictability of models. The significance of threading template alignments and the convergence parameters of structure assembly simulations are used to compute the C-Score. The standard C-score range is −5 to 2, with a higher C-score indicating a model with high confidence. The putative protein structure was selected based on the predicted 3D model with the highest C-score.

### 2.4 General CRISPR target design and generation of short guide RNA

Primers and single-guide RNA (sgRNA) sequences were designed by considering genomic variation and using the known genetic variation of the GRCz11 annotation (ENSDARG00000036155). Primers, single guide RNA (sgRNA), tracrRNA, and Cas9 Nuclease V3 were purchased from Integrated DNA Technologies BVBA (IDT, Leuven, Belgium). The CHOPCHOP web-tool [42] was used for both GN or NG as 5′ specifications. The Supplemental table displays primers and sgRNA sequences (Sup. 1).

### 2.5 Generation of mutant lines

Zebrafish *GLA* mutants were generated by CRISPR/Cas9-mediated gene tool. One-target region located within *GLA* exon five was chosen for sgRNA recognition. The corresponding sgRNA was injected into wild-type zebrafish embryos (n = 200) at the 1-cell stage together with cas9 protein. For mutation screening, sgRNA-injected embryos (Founder 0/F_0_) of 5 dpf were screened by PCR fragment analysis to confirm successful mutant generation. After validating the successful F_0_ mosaic generation, larvae were raised to adulthood, screened individually, and positive mutants were out-crossed to TAB wild-type adults to obtain the first generation F_1_ embryos with heterozygous genotype.

To obtain the *GLA*^−/−^ mutant line, selected F_1_ adult were in-crossed and produced the second generation F_2_ in which the progeny was *GLA*^+/+^, *GLA*^+/−^ and *GLA*^−/−^. Homozygous *GLA*^−/−^ mutant individuals from F_2_ were genotyped, sequenced, and in-crossed to generate third generation F_3_, 100% homozygous *GLA*^−/−^ mutant. All work has been done on F_3_ generation or its in-crossed progeny compared to the wild type of similar crossing batch Sup.2.

### 2.6 Genotyping and sequencing

Genotyping was performed using simplified PCR fragment analysis. To genotype *GLA* mutants, genomic DNA was extracted from either whole embryos/larvae or the tail fin of adults with the DNA crude extraction method. Briefly, samples were collected in 50 μL of 50 mM NaOH, samples were then heated at 95 °C for 20 min followed by quick cooling at 4 °C. Genomic DNA was used as template for genotyping PCR. The PCR was carried out at the following conditions: 95 °C for 5 min; 35 cycles of 94 °C for 30 s, 58 °C for 30 s, 72 °C for 30 s, and final elongation step of 72 °C for 5 min. PCR product was digested with restriction enzyme *Bsm*FI (R0572L), New England BioLabs, Ipswich, MA, USA) 65 °C for 1 h, following manufacturer instructions. Final digestion products were resolved on 2% agarose gels.

For sequencing, PCR products were cleaned up using ExoSAP-IT™ (Applied Biosystems™), following manufacturer instructions. Sequencing reactions were prepared following BigDye v.3.1 Protocol, Sequencing Facility, High Technology Center, UiB, with the following sequencing cycle: 96 °C for 5 min; 25 cycles of 96 °C for 10 s, 58 °C for 5 s, 60 °C for 4 min. Automated Sanger DNA Sequencing was performed using capillary based Applied Biosystem 3730XL Analyzer.

### 2.7 Alpha-galactosidase A activity assay and lysoGb3 measurement

To compare α-GAL activity levels at different developmental stages, samples were collected from 2dpf embryos (n = 7), 4dpf larvae (n = 8), and 30dpf juveniles (n = 4). For +90 dpf adults, kidney samples were pooled (3/sample, male n = 10, female n = 9). For a comparison between adult wild type and mutant, kidney lysates from each gender were sampled separately (n = 10). Samples were snap-frozen in liquid nitrogen immediately after collection and shipped in dry ice to Sahlgrenska University Hospital, Sweden, to measure α-GAL activity and lysoGb3 levels. Samples were stored at −80 °C upon arrival and before the analysis.

For α-GAL activity analysis, samples were kept on ice, diluted in 100–200 μL of deionized water, depending on tissue sample weight, and homogenized at 4 °C (glass/Teflon; 10–15 strokes). Protein concentration was determined by the BCA Protein Assay Reagent method (Pierce, Rockford, USA). Enzyme activity was assessed using the α-GAL standardized protocol used in clinical diagnostics [43], [44]. Briefly, the activity of tissue homogenates was measured using a fluorometric assay utilizing 4-metylumbelliferyl (MU)-alpha-galactopyranoside (20 mM, Apollo Scientific) as substrate. Sodium acetate buffer (0.1 mM, pH 4.5) was used as substrate buffer. The substrate solution included *N*-acetyl-D-galactosamine (200 mM, Toronto Research Chemicals) as an inhibitor of alpha-*N*-acetyl-galactosamidase (previously named alpha-galactosidase B), as well as 2% delipidized bovine serum albumin. The enzyme reaction was performed at 37 °C, pH 4.5 for 30 min, and stopped with glycine buffer (0.25 M, pH 10.3, Merck). Fluorescence of samples, blanks, and standard solution (4-Methyl Umbelliferon 1 μM, diluted in Glycine buffer) was measured by spectrofluorometry (Jasco FP-6500, Jasco Inc., Easton, MD, USA) using an excitation wavelength of 360 nm and an emission wavelength of 448 nm. The activity of α-GAL was expressed as μkatal/kg protein.

Analysis of lysoGb3 was performed in tissue homogenates of zebrafish kidney by a modified method, as previously described [45]. Samples were prepared as described above, and 50 μL of the homogenate was used for analysis.

### 2.8 Customized polyclonal antibody synthesis

Alpha-galactosidase protein sequence was screened using Geneious prime software for potential antigenic regions using the default setting of the software. The minimum length of the antigenic region was set to 7 amino acids. The antigenic region prediction was powered by the EMBOSS6.5.7 tool plugin within Geneious prime software. The selected sequences were then investigated using BLAST against rabbit sequence for sequence similarities to avoid cross-reactivity. Moreover, GenScript® proprietary software evaluated their antigenicity. Accordingly, two antigenic sequences were selected for the rabbit-generated polyclonal antibody against zebrafish α-GAL protein. Both antigenic peptides were at the seventh exon of the protein. The two antigenic peptides of the carboxy-terminal amino acids 251–264 [(NH_2_-) CKADSFELWERPLSG (-CONH_2_)] and 271–284 [(NH_2_-) CVVNRQEIGGPRRFT (-CONH_2_)] were synthesized and coupled to KLH carrier *via* the cysteine residue (underlined). Antibodies were then produced by GenScript's standard protocol in New Zealand rabbits.

### 2.9 Plasma creatinine assessment

Zebrafish were euthanized by immersion in tricaine methanesulfonate MS-222300 mg/L, Sigma Chemical Co., St. Louis, Mo. (Cat. No. A-5040). Blood was immediately collected from the dorsal aorta, as described [46]. Briefly, a transverse cut was made just caudal to the dorsal fin. Blood spilling from this cut was rapidly collected by a heparin-coated micropipette tip and pooled to achieve the desired volume of 20 μL (n = 2/genotype).

Plasma concentrations of creatinine were measured by high-performance liquid chromatography/tandem mass spectrometry following [47] in collaboration with Bevital AS, Bergen, Norway.

### 2.10 Proteinuria assessment

In humans, albuminuria and proteinuria are standard clinical methods to evaluate kidney function. However, zebrafish does not produce albumin, therefore, albuminuria cannot be detected. We adopted a new method to qualitatively assess the pattern of proteinuria in zebrafish adults. For each measurement, one wild-type and one mutant fish were kept in 100 ml water for 24 h at 28.5 °C. Water samples (n = 5) were harvested after ensuring that the fish was alive, and blank was used as a negative control (only water). Protein was precipitated using Trichloroacetic acid (TCA): Chloroform method. Briefly, 100% TCA solution was added, mixed gently with the water sample and incubated at 4 °C for 30 min. Samples were then centrifuged at 13,000 rpm at 4 °C for 5 min and supernatants were discarded. Pellets were washed 2× with cold acetone, with each washing step followed by centrifugation at 13,000 rpm at 4 °C for 5 min. After drying the pellets, 20 μL of sample buffer was added and the mixture was incubated at 70 °C for 10 min. For SDS-PAGE, the samples were applied to NuPAGE 4–12% Bis-Tris Gels (Invitrogen). The gel was stained and destained with Coomassie Brilliant Blue R-250 Staining Solution kit, Cat#1610436, and imaged using ChemiDoc XRS+ system, BIO-RAD.

To identify protein/s in each gel, bands were excised using a clean blade under sterile conditions. Cut gel bands of similar sizes were placed into a sterile 70% ethanol-pre-cleaned 1.5 mL tube. Sufficient sterile water was added to cover excised gels. and samples were kept at —20C until being shipped to Department of Biosciences, University of Oslo, where they were analyzed using LC-MS/MS, detailed method below.

#### 2.10.1 In-gel protein digest

Gel slices containing proteins were destained, reduced, alkylated, and digested with trypsin (Sigma) as previously described [37].

#### 2.10.2 LC-MS/MS analysis of protein fractions

The generated peptide samples were analyzed using an Ultimate 3000 nano-UHPLC system (Dionex, Sunnyvale, CA, USA) connected to a QExactive mass spectrometer (ThermoElectron, Bremen, Germany) equipped with a nano electrospray ion source. For liquid chromatography separation, an Acclaim PepMap 100 column (C18, 3 μm beads, 100 Å, 75 μm inner diameter, 50 cm) (Dionex, Sunnyvale CA, USA) was used. A flow rate of 300 nL/min was employed with a solvent gradient of 3–55% B in 53 min, to 96% B in 2 min and retaining that for 5 min then back to 3% B in 3 min. Solvent A was 0.1% formic acid and solvent B was 0.1% formic acid/90% acetonitrile. The mass spectrometer was operated in the data-dependent mode to automatically switch between MS and MS/MS acquisition. Survey full scan MS spectra (from *m*/*z* 200 to 2000) were acquired with the resolution *R* = 70,000 at m/z 200, after accumulation to a target of 1e6. The maximum allowed ion accumulation times were 100 ms. The method used allowed sequential isolation of up to the ten most intense ions (intensity threshold 1.7e4), for fragmentation using higher-energy collision induced dissociation (HCD) at a target value of 10,000 charges and a resolution *R* = 17,500 with NCE 28. Target ions already selected for MS2 were dynamically excluded for 60 s. The isolation window was m/z = 2 without offset. The maximum allowed ion accumulation for the MS/MS spectrum was 60 ms. For accurate mass measurements, the lock mass option was enabled in MS mode for internal recalibration during the analysis.

#### 2.10.3 Database search and label-free quantitation

Data were acquired using Xcalibur v2.5.5 and raw files were processed. Database searches were performed against the Zebrafish (*Danio Rerio*) (NCBI; taxon ID7955; 55,761 entries) and the PD common contaminants list, with the proteome discoverer v 2.4 software (ThermoScientific, Whaltham, Massachusetts, USA). The following parameters were used: digestion enzyme, trypsin; maximum missed cleavage, 2; minimum peptide length 4; parent ion error tolerance, 10.0 ppm; fragment ion mass error tolerance, 0.04 Da; and fixed modifications, carbamidomethylation of cysteines. Oxidation of methionine and acetylation of the N-terminus were specified as variable modifications and the maximum number of PTMs was set to 2. Peptide-spectrum matches was assessed with percolator with FDR target set at 0.01 (strict) and 0.05 (relaxed). Generated protein lists were manually curated, with low FDR proteins, proteins with single (low score) peptides, and contaminants removed. Functional annotation of proteins was done using the PD protein knowledge-database linked to GO: annotations.

### 2.11 Histology and immunohistochemistry

For standard histology and immunohistochemical staining, wild type (n = 6) and mutant (n = 3) adult (90 + dpf) zebrafish were euthanized in 300 mg/L tricaine methanesulfonate MS-222 Sigma (Cat. No. A-5040) and dissected open. Kidneys were exposed after discarding viscera under 1× PBS (Life Technologies, Cat. No. AM9625). Dissected fishes were fixed with 4% paraformaldehyde in 1× PBS for 48 h. Later, kidneys were removed, processed, and embedded in paraffin according to standard protocols of molecular imaging center MIC, UiB and Pathology Department, Haukeland University Hospital. Sections of 5 μM were acquired for histology (Hematoxylin and periodic acid Schiff stain) and immunohistochemical staining.

Immunohistochemistry was performed as previously described [48] with slight modifications. Antigen retrieval was skipped upon IHC protocol optimization. Staining with our customized rabbit polyclonal primary antibody (anti-α-GAL) was performed for one hour at room temperature at 1:600 concentration. For negative controls, the primary antibody was omitted. Slides were scanned with ScanScope XT® (Aperio) at x40 resulting in a resolution of 0.25 μm per Pixel. Digital slides were viewed in ImageScope 12.

Immunohistochemical positivity for anti-α-GAL was quantified using the color deconvolution algorithm version 9.1 (Aperio, CA, USA) after adjusting the default parameters to DAB staining. The total percentage of positive pixels was used as a visualization parameter and statistics was performed IBM SPSS V. 25.

For transmission electron microscopy (TEM), kidney samples of wild type (n = 9) and mutant (n = 8) were fixed overnight at 4 °C in 2.5%. Samples were washed 3 times in 0.1 M cacodylate buffer and then incubated for 1 h in 1% osmium tetraoxide and washed with cacodylate buffer. Samples were dehydrated in ascending ethanol concentrations and incubated in ethanol and propylene oxide (PO). Samples were then infiltrated with 25% Epon 812 resin and 75% PO for 35 min, followed by 50% Epon 812 resin and 50% PO for 1 h and exchanged with a new 50% Epon 812 resin and 50% PO for overnight incubation. Samples were exchanged with 75%: 25% (resin: PO), then pure epoxy resin for 3–4 h, then overnight. Finally, the resin exchanged completely with epoxy resin for 3 h, embedded in epoxy resin, and polymerized at 60 °C for 48 h.

Sections 500 nm to 1 μm thick were collected using a Leica ULTRA-CUT microtome. Thick sections were stained with 1% toluidine blue. 70–80 nm ultrathin sections were cut from this block, collected on 300-mesh copper grids, and stained with 2% uranyl acetate (aqueous) for 16 min and then with lead citrate for 12 min. Samples were imaged at MIC, Department of Biomedicine, UiB, on the Jeol JEM-1230 electron microscope at various magnifications.

#### 2.11.1 Measurement of podocyte foot process width

Glomerular basement membrane GBM length was measured using Fiji (ImageJ) [49]. The number of podocyte foot processes along the GBM was counted manually. Any connected epithelial segment laying on GBM and separated by filtration slit was considered as a foot process. The arithmetic mean of the foot process width was calculated from the following equation:

$$\mathrm{FPW}=\frac{\pi }{4}∙\frac{\sum \mathrm{GBM}\;\text{length}}{\sum \mathrm{FP}\;\mathrm{No}.}$$

Where ∑FP No. is the total number of foot processes counted in each picture, and ∑GBM length is the total GBM length measured in each picture. The hypothetical random variation in the angle of section relative to the long axis of the podocyte was corrected using the correction factor π/4. Mean width was calculated first for an individual sample, and then it was used to calculate the mean FPW for the two groups (wild type and mutant). 1–3 glomeruli were evaluated per fish (n = 9 wildtype, n = 8 mutant).

### 2.12 Statistical analyses

Statistical analysis was performed using IBM SPSS V 25 and GraphPad Prism V 9.2.0. Values are presented as violin plots (median/interquartile ranges) or as mean ± SD. The Kruskal–Wallis test with Dunn test for *post hoc* comparison or Mann-Whitney test were used to assess statistical significance. Differences were considered significant with p-values <0.05.

## 3 Results

### 3.1 Zebrafish *GLA* is the only orthologue for its human counterpart

To develop an innovative experimental model of potential relevance for the study of human FD, we first investigated the expression of *GLA* homologues in zebrafish.

We found only one previously annotated version of the human *GLA* gene counterpart in zebrafish (chromosome 14: NC_007125.7) in the National Centre for Biotechnology Information (NCBI) online databases, named galactosidase, alpha. This gene, similar to its human counterpart, is composed of seven exons, encoding a 1470 bp mRNA (NM_001006103). A polypeptide homologous to human α-GAL protein was also identified (NP_001006103.2).

To explore their exclusive homology to the human gene and protein counterparts, we used the nucleotide (NC_000023.11) and the amino acid (NP_000160.1) sequences of human GLA gene and α-GAL protein as queries in non-redundant sequence databases from NCBI, against the Zebrafish May 2017 (GRCz11/danRer11). Indeed, zebrafish *GLA* gene mRNA is highly similar to its human counterpart (71% bp sequence identity). Moreover, a comparison between zebrafish and human proteins revealed a high degree of sequence similarity (65.53% amino acids identity). Most importantly, active, and substrate-binding sites are 100% conserved along with the primary structure of zebrafish protein.

**Fig. 1:**
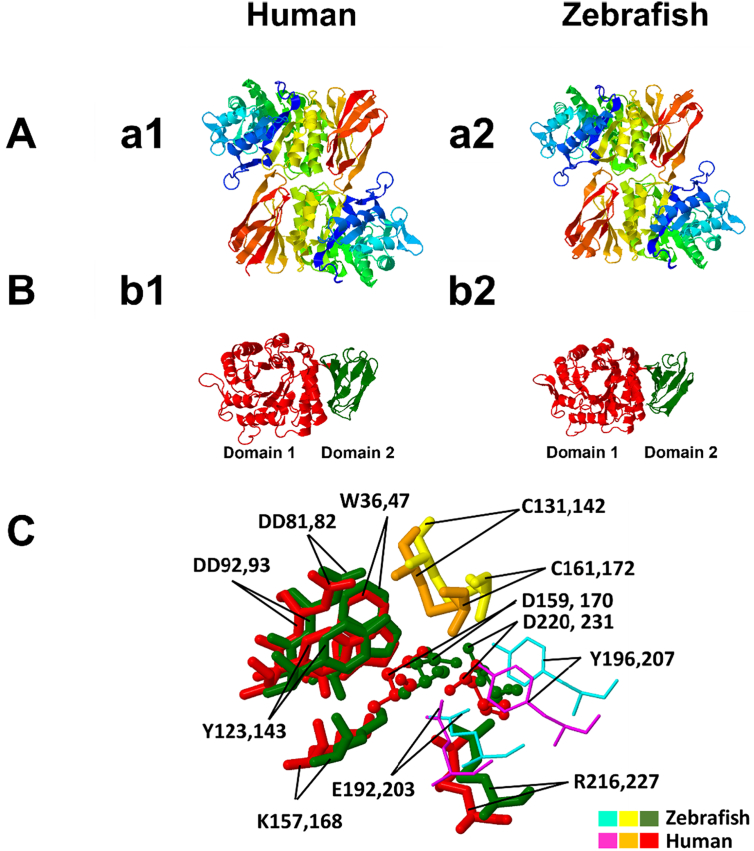
Structure prediction of the zebrafish α-GAL. (a1) homodimer structure of human α-GAL; (a2) homodimer structure of zebrafish α-GAL. The dimers are colored from N-terminal (blue) to C-terminal (red). The structure prediction shows that the zebrafish α-GAL folds in a pattern highly similar to its human counterpart. (b1) is the monomer of a human α-GAL enzyme showing the two domains, while (b2) is the zebrafish α-GAL enzyme showing the same structure. Domain 1, (β/α)8 barrel, extends through residues 32–330 in human (red) and 21–319 in zebrafish (red), and contains the active site, while domain 2 extends through residues 331–429 in human (green) and 320–409 in zebrafish (green) and contains antiparallel β strands. C: the superimposed structure of the two enzymes active sites and substrate binding site, with substrate specificity residues W36, D81, D82, Y123, K157, R216 in zebrafish and W47, D92, D93, Y143, K168, R227 in human, are fully conserved between the two species. The active sites D159 and D220 in zebrafish, D170, and D231 in humans are also conserved. The residues C131 and C161 zebrafish and C142 and C172 human help to stabilize the conformation of the substrate-binding site through the formation of disulfide bonds. The substrate-binding site boundaries E192 and Y196 in zebrafish correspond to E203 and Y207 in humans. Color ligands for zebrafish and human are shown in plate C. (For interpretation of the references to color in this figure legend, the reader is referred to the web version of this article.)

Based on this background, we performed a homology modeling analysis on I-TASSER online modeling service, and we evaluated associated C-scores. After removing the first 20 amino acid residues comprising the signal sequence, the structure revealed was similar to its human counterpart, *e.g.* a homodimeric protein (Fig. 1 a1 and a2) with each monomer composed of two domains (Fig. 1 b1 and b2), first, a N-terminal (β/α)_8_ domain (residues 21 to 319), containing the active site, and second, a C-terminal domain (residues 320 to 409), containing antiparallel β8 strands packs against the first domain.

As expected from the high protein sequence similarity, the model fits well on the template when superimposed with Geneious prime software. The positions of the catalytic residues Nucleophile (159D) and Proton donor (220D) together with the substrate-binding site 192E are fully conserved (Fig. 1 C). Moreover, the position of the N-glycosylation site (N181), and the Ubiquitination sites (K116, 229, 297, 303, and 315), and the five-disulfide bond organization (C41–C83, C45–C52, C131–C161, C191– C212, and C367–C371) are similarly conserved. Among these, C131-C161 is the most important one, as it is directly involved in stabilizing the conformation of the active site. Also, amino acids, W36, D81, D82, Y123, K157, and R216 conferring substrate specificity for α-GAL are in topologically conserved positions (Fig. 1 C). The non-conserved residues between mammalian and zebrafish α-GAL enzymes are exposed on the surface of the predicted zebrafish α-GAL structure.

This data indicates that *GLA* is the only zebrafish orthologue for the human *GLA* gene and encodes a protein with high *in silico* similarity to its human counterpart.

### 3.2 Zebrafish-α-GAL tissue distribution and functional activity are similar to their human counterpart

The kidney is a major affected organ in classical FD in humans. To investigate the pattern of α-GAL enzyme distribution, as related to its human counterpart, immunohistochemical analysis of adult zebrafish kidneys was performed by using a customized polyclonal antibody. Worth mentioning here that only one antigenic peptide was able to produce an appropriate antibody against zebrafish α-GAL, that is amino acids 251–264 [(NH2-) CKADSFELWERPLSG (-CONH2)].

**Fig. 2:**
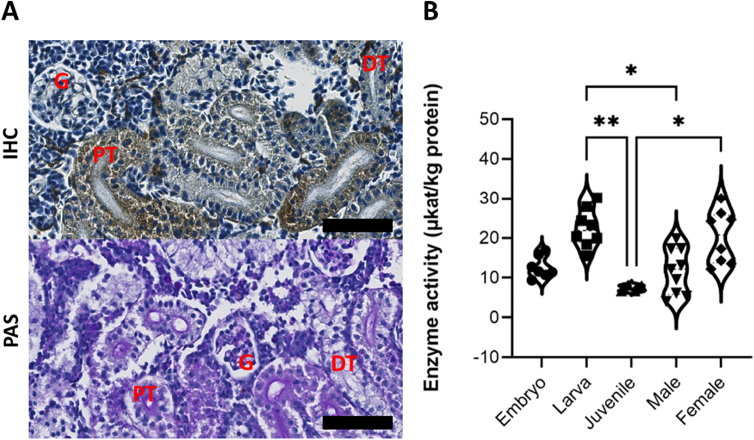
Kidney tissue distribution and enzyme activity in wild type zebrafish. (A) Immunohistochemistry (IHC) staining for Zebrafish α-GAL in wild-type adult zebrafish kidney. The expression pattern is similar in both genders. The protein was abundantly detectable in the cytoplasm of renal tubule cells, and to a lower extent in the glomerulus. Hematoxylin was used as counterstain. The lower panel shows the three renal structures, as stained with Periodic Acid Schiff (PAS). Scale bar (in black) = 50um. (B) Wild-type zebrafish α-GAL activity as evaluated in the whole embryo, larvae, and juvenile whole body tissue lysates and in adult male and female kidney tissue lysates (Kruskal-Wallis, P ≤ 0.05). Results of the Dunn *post hoc* test show a significant difference (*) between juvenile and larvae (P < 0.05) and juvenile and adult female specimens (P < 0.05). The violin plot represents these results within the 95th percentile. G = glomerulus, PT = proximal tubule, DT = distal tubule. Validation of the antibody is provided in Sup.4.

Distribution of the α-GAL enzyme immune-reactivity was limited to the cytoplasm of podocytes and proximal and distal tubule cells. However, while in the podocytes, the α-GAL signal was scarcely distributed in the cytoplasm, it was extensively present in the renal tubules (Fig. 2 A). Thus, immunohistochemical results indicate that the α-GAL distribution in the zebrafish's kidney is similar to that in the human kidney [50].

The sole presence of the protein does not necessarily reflect the same functional activity as in humans, where its deficiency is crucial in determining FD manifestations. We therefore evaluated the enzymatic activity of zebrafish α-GAL in the animal tissue lysates (embryo, larva, and juvenile) and in kidney lysates of adult males and females. A comparative analysis revealed a significant difference between larva and juvenile stages (p = 0.006), larva and adult male (p = 0.03), and between juvenile and adult female (p = 0.048) (Fig. 2 B).

These results not only demonstrate the presence of zebrafish α-GAL throughout different developmental stages, but also validate the functional similarity of zebrafish α-GAL and its human orthologue by utilizing the same chemical substrate. Thus, due to our in-depth bioinformatic analysis demonstrating high levels of identity on both sequence and structural levels coupled with our enzymatic *in-vitro* assay demonstrating functional similarity to human α-GAL we hereafter refer to NC_007125.7 as zebrafish α-GAL.

We additionally measured lysoGb3 in zebrafish kidney tissue lysate but obtained negative result Sup. 3. These results are in line with previous work [33] and supporting our *in-silico* observation that zebrafish lacks Gb3 synthase gene.

### 3.3 Generation and verification of the mutant line

**Fig. 3:**
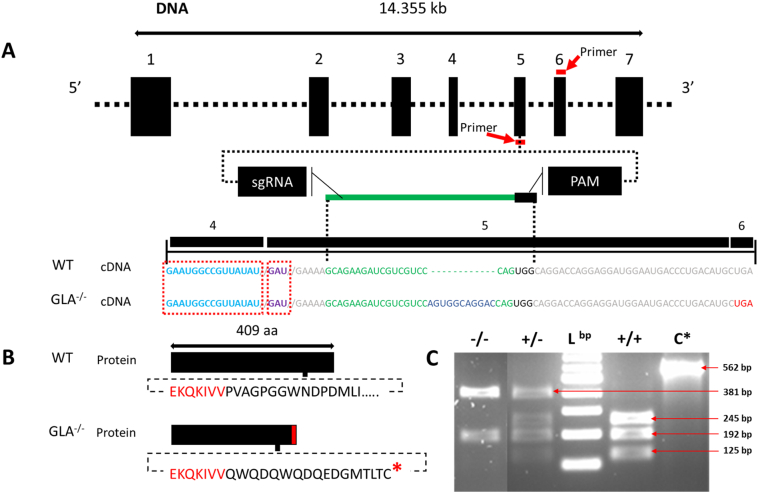
Generation and verification of a GLA mutant line. (A) CRISPR/Cas9-based gene-editing tool used to generate mutant zebrafish. The targeted genomic sequence for the introduction of the insertion mutation in the exon 5 is illustrated. The eleven base pair insertion (shown in blue) is highlighted in the cDNA sequence. The putative stop codon after the insertion is highlighted in red. Guide RNA and PAM sequences are highlighted in green and black, respectively. Mutation occurred 45 bp from the proton donor catalytic site (in violet) and 120 bp from the substrate binding site (in light blue) that is in exon 4. Primers for genotyping and sequencing spanning exons 5 and 6 are indicated by the red bar (B) The insertion mutation leads to a frameshift with a premature stop codon (in red) 16 amino acid downstream the insertion region (the lower panel). (C) PCR screen shows that the insertion interrupts one of the two restriction enzyme digestion sites resulting in two PCR fragments in the mutant (−/−) instead of three in the wild type (+/+), L^bp^ = DNA ladder in base pairs (bp), C* = wild type undigested PCR product (the agarose gel image is edited for illustration, full image can be reviewed in sup.5). (For interpretation of the references to color in this figure legend, the reader is referred to the web version of this article.)

To investigate “*in vivo*” the effects of reduced α-GAL enzyme production in zebrafish, we used CRISPR/Cas9 gene-engineering tool to edit the *GLA* gene. Sequencing of the edited line revealed a 11 bp insertion in exon 5 (the targeted region) at moderate proximity to the active site (Fig. 3). The mutation was 45 bp downstream from the proton donor catalytic site in exon 5 and 120 bp downstream from the substrate binding site, in exon 4 (Fig. 3). The insertion resulted in a frameshift started at position c.802, p244.P > Q, and a subsequent premature stop codon within exon 6 at c.842, p.261.I > *.

### 3.4 *GLA* indel mutation results in decreased enzymatic activity and expression of zebrafish α-GAL in renal tissues

**Fig. 4:**
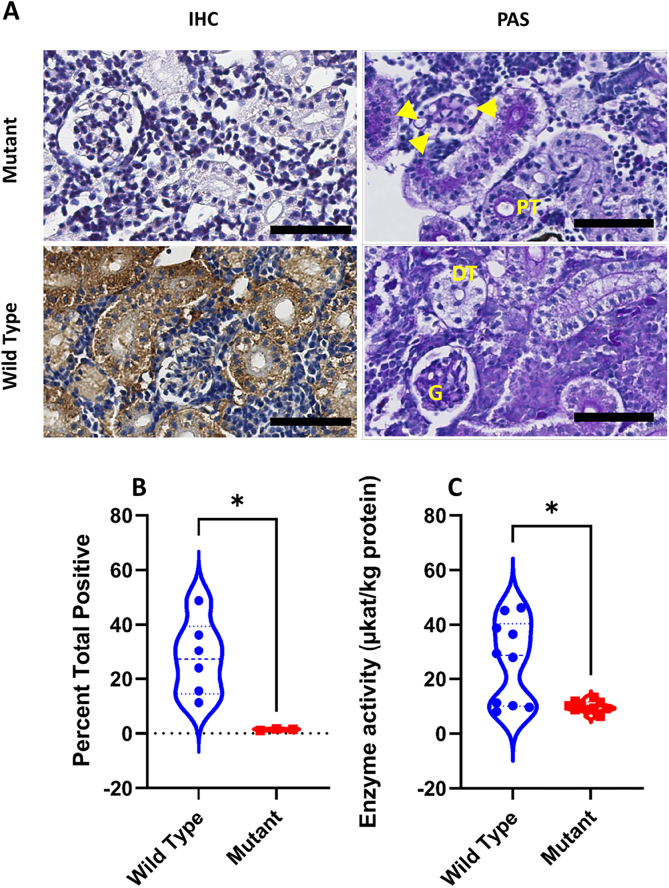
Histological distribution and enzymatic activity of Zebrafish- α-GAL protein in the GLA mutant zebrafish. (A) Scanned digital images show α-GAL antibody and periodic acid Schiff staining. Note that PAS staining shows dilated capillary loops and thinner Bowman's space (yellow arrowheads) in mutant compared to wild type animals. (B) Quantification of immunohistochemical analysis of sections from wild-type and mutant kidneys. Signal intensity is significantly higher in wild type compared to mutant tissues (Mann-Whitney *U* test: P < 0.05). The violin plot represents these results within the 95th percentile, and the dash-line symbol inside the violin plot represents the median. Scale bar = 50um. (C) Comparison of α-GAL activity in wild type and mutant zebrafish kidney tissue lysate (Mann-Whitney Test, P < 0.05).

DNA sequencing analysis indicates that the mutation introduced generated a frameshift in the resulting mRNA. To validate these results at the protein level, we measured the enzyme activity in the kidney tissue lysate of mutant animals and their wildtype counterpart. Our results revealed an approximately 65% decrease of enzyme activity in the mutant fish, as compared to the wild type (p = 0.025) (Table 1, Fig. 4).

**Table 1:** The α-GAL enzyme activity measurements in μkat/kg of protein in wild type and mutant. N:sample number; SD: standard deviation.

	N	Mean ± SD	Median	Mean Rank
Wild type	10	26.3 ± 15.4	28.60	13.45
Mutant	10	9.9 ± 1.9	9.700	7.55

Based on the enzyme activity data, we anticipated a reduced presence of the enzyme in the renal tissue. Therefore, we performed immunohistochemical analysis to quantify α-GAL protein expression in wild-type and mutant renal tissue. We observed that no or very weak α-GAL signal was detectable in mutant, as compared to wild type renal tissue (p = 0.024, Fig. 4). We also used periodic acid Schiff (PAS) stain to document glomerular changes and we observed dilated capillary loops and thinner Bowman's space (Fig. 4) in the mutant compared to the wild type kidneys.

### 3.5 Compromised renal function in *GAL* mutant zebrafish

**Fig. 5:**
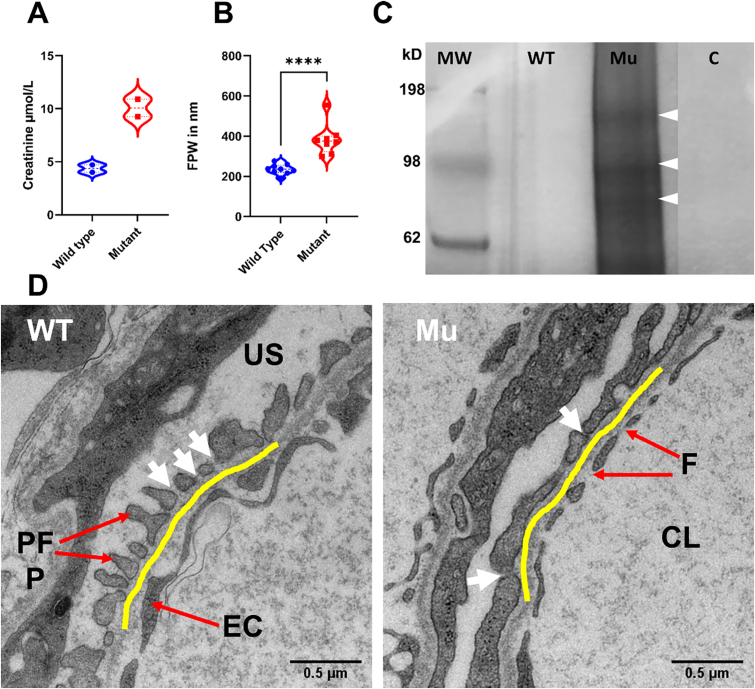
GLA-mutant zebrafish show compromised filtration barrier function. (A) Renal impairment is documented by double plasma creatinine level in the mutant zebrafish. (B and D) FPW quantitation in adult zebrafish kidney on TEM images reveals wider foot processes (podocyte foot process effacement) in the mutant (Mann-Whitney Test, P < 0.05. Violin plots depict results within the 95th percentile. The yellow line highlights the GBM. White arrows point to the silt diaphragm between two adjacent foot processes. CL = capillary lumen, F = fenestrae, EC = endothelial cell, US = urinary space, PFP = podocyte foot process. (C) Renal function impairment is further validated by the leakage of high molecular weight proteins observed by SDS-PAGE electrophoresis. The illustrated protein bands in the fig. (80 kDa, 98 kDa and 150 kDa wight arrowheads) were identified by mass spectrometry analysis. MW = marker in kDa, WT = wild type, Mu = Mutant, C = control (water). The polyacrylamide gel image is edited for illustration, full gel image can be reviewed in Sup.7.

Same as in humans, in zebrafish creatinine is freely filtered through the glomerulus. Therefore, plasma creatinine level mirrors the integrity of the glomerular barrier. We assessed the plasma creatinine of wild type and mutant zebrafish. We observed more than double volume plasma concentrations of creatinine in mutant compared to wild type animals (mean SD) in μmol/L: 10.08 ± 1.167 *vs.* 4.36 ± 0.4950 (Fig. 5 A), consistent with a severely compromised kidney function.

Accordingly, we were also interested in evaluating impaired filtration barrier integrity using zebrafish-adapted proteinuria assessment. Indeed, a high molecular weight protein leakage was detectable in mutant, compared to the wild type fish (Fig. 5 C). The main high molecular weights proteins crossing filtration barrier were detected at 80 kDa, 98 kDa and 150 kDa. These proteins were identified by mass spectrometry after individual bands were excised from polyacrylamide gels (Sup.6). Interestingly, the yolk-transport protein abundantly found in blood, vitellogenin, was also detectable in all three gel bands. This could be explained by non-specific protein fragmentation [51]. This protein is the zebrafish counterpart of human plasma albumin [52] and we therefore interpreted this as a valuable test to assess the integrity of the membrane-filtration barrier.

To further validate our results at the TEM level, we measured podocyte foot process width (FPW) (Fig. 5 B). In zebrafish, no reference or standard FPW is available. However, previous studies [53] suggest that in normal state, it is half-sized, compared to its human counterpart (508–827 nm) [54]. Accordingly, within GBM distances ranging between 21,000 and 207,000 nm (average total glomerular capillary circumference: 22157 nm), FPW ranged between 192 and 276 nm (235.5 ± 24.76 nm) in wild type but between 298 and 555 nm (383.4 ± 78.43 nm) in mutant animals (P < 0.05) (Fig. 5 B).

## 4 Discussion

Innovative supplemental therapeutic approaches for FD are urgently needed. However, *in vitro* and *in vivo* models currently used to test novel treatments, fail to adequately mirror FD pathobiology. Here, we have developed a zebrafish-based experimental model to allow a reliable and rapid *in vivo* assessment of the potential clinical relevance of new molecules and biologicals and to elucidate non Gb3-dependant pathophysiological disease mechanisms.

*In-silico* investigation demonstrated the presence of a single orthologue for human *GLA* gene in zebrafish. Furthermore, structure prediction revealed a high similarity between human and zebrafish α-GAL (Fig. 1), this is in agreement with previous report [55]. Importantly, by using a polyclonal antibody against zebrafish α-GAL we could show that the distribution of α-GAL in zebrafish kidneys closely resembles that of its human counterpart (Fig. 2 A). Most interestingly, the human α-GAL activity assays can effectively be used to measure zebrafish enzyme activity and we could therefore demonstrate that it is present in zebrafish as early as in 2 dpf embryo, 4 dpf larva, 30 dpf juvenile and adult fish tissues 90^+^ dpf (Fig. 2 B), and fluctuates similarly to humans [56]. We have noticed that enzyme activity in general is higher in adult wildtype female compared to adult male zebrafish, in agree with what have been described previously in Fabry mice [57] as well as in human [58]. For both Fabry mice and human this could be explained by the X-chromosome inactivation patterns [59], however, in zebrafish, as the *GLA* gene is not X-linked, we can attribute the enzyme activity difference to undetermined endogenous gender-related factors, *i.e.* gender-related expression pattern [52]. In fact, variable reports have indicated that individuals with FD disease are known for having normal values of the enzyme activity in response to variable mutations [60], [61], [62] while the lysoGb3 maintain elevating [63]. Apart from this, enzyme activity *in vitro* can differ from *in vivo* activity for many factors, *i.e.* the nature of the used substrate [64], [65]. No difference was observed in the size or the weight between mutant and wildtype fish, however, we have observed higher mortality rates during the early embryonic stages, particularly when the crossing is made between younger mutant generations e. the 5th generation (F4) see Sup.8.

To provide a functional validation of our data, using CRISPR/Cas9 gene-editing tool, we introduced a hypomorphic mutation in exon 5 of *GLA* gene, resulting in decreased α-GAL activity. Sequence analysis indicates that the eleven base pair insertions at the end of the 5th exon and close to the active site (Fig. 3, Sup.9), resulted in a frameshift and introduced a premature stop codon at the beginning of the 6th exon. Notably, this mutation resembles a previously described rs869312402 c.785G > A-Trp262* mutation, which is pathogenic and leads to a classical FD phenotype in humans [66], [67].

Most importantly, in animals bearing the engineered *GLA* gene, expression of the specific gene product in the kidneys was nearly undetectable by immunohistochemistry (Fig. 4 A and B), and enzyme activity was markedly reduced (Fig. 4 C).

During FD treatment, evaluation of proteinuria and serum/plasma creatinine is crucial to monitor disease progression and treatment outcome. Accordingly, we observed that plasma creatinine is higher in mutant, compared to wild type zebrafish. Moreover, proteinuria assessment unraveled a leak of high molecular weight proteins in the mutant fish. Combined, these observations support a glomerular filtration impairment. In agreement with this data, microscopic examination of kidney sections revealed increased glomerular size in mutant zebrafish characterized by dilated capillary loops, enlarged glomerular diameter, and thinner Bowman's space (Fig. 4 A). Similar findings have been reported in human Fabry nephropathy [68], [69], [70], [71] but not in other FD model [29], [72].

Increased podocyte and glomerular volumes have recently been described in classical FD patients by non-biased ultrastructural methods [73]. In accord with these reports, ultramicroscopic investigation revealed significant podocyte foot process effacement in the zebrafish mutant line.

Taken together, these results are consistent with the general assumption that damage of the kidney filtration barrier results in protein leakage and elevated plasma creatinine which are critical in defining glomerular filtration rate loss [74], [75]. Most importantly, similarities with human FD phenotype not associated with Gb3 accumulation support the important role of substrate independent pathophysiologic mechanisms in Fabry nephropathy [76].

Limitations of the proposed model should be acknowledged. In particular, FD is characterized by a slow systematic accumulation of Gb3 [9], [77], [78] but zebrafish does not have Gb3 synthase gene, and hence no Gb3 accumulation can be expected. However, in FD, proteinuria represents an early clinical sign of kidney damage [79], [80], even before Gb3 accumulation in the podocyte [79], [81], and low range proteinuria may start as early as at 5 to 10 years of age [82], [83]. Therefore, it is widely recognized that apart from Gb3, in humans, multiple and not fully elucidated signal mechanisms do work in concert with histopathologic mechanisms [84]. Nevertheless, the possibility to insert Gb3 synthase gene or to transiently express it in zebrafish could be considered so that we can evaluate the effect of GLA inactivation on Gb3 *in vivo* and establish the hallmark of FD, Gb3 accumulation.

Notably, a residual enzyme activity was also detectable in mutant animals. It is worth mentioning that this residual activity could be due to α-*N*-acetylgalactosaminidase (α-galactosidase B) since the inhibitor (*N*-acetyl-D-galactosamine) concentration is optimized for human samples. Nevertheless, the low immunohistochemically detected signal of zebrafish α-GAL may compromise this assumption. Since α-GAL protein is remarkably undetectable by IHC in the mutant line, yet partially functioning, we speculate that the generated stop codon might be to some extent bypassed during protein translation, leading to structural deformation/protein misfolding with residual enzymatic activity.

On the other hand, the zebrafish model presents several advantages. Previously established Fabry mice, appear to be clinically normal, with no abnormality in blood and urine analyses and a normal lifetime. Kidney damage is only detectable by histological analysis in 20 weeks old mice (young adult) [32], [85]. However, this could be achieved by day five post fertilization in zebrafish, thus in a considerably shorter time.

Besides the lack of the Henle loop, zebrafish and human kidneys are highly similar, sharing all fundamental functional units, and, especially, podocytes [22]. Moreover, expression pattern of zebrafish α-GAL is analogous to humans and enzyme activity can be easily and consistently measured throughout all developmental stages.

Another distinct advantage of zebrafish is represented by non-invasive drug administration protocols. Whether through an aqueous environment or oral gavage, this allows feasible drug delivery for both embryos and adult fish with reduced stress, thereby allowing more accurate observation of the histo- and physiopathology in the fish [86], [87]. Additionally, progress in high-throughput drug screening in zebrafish has recently been documented [88].

Our results not only provide strong evidence for a structural and functional similarity of human and zebrafish α-GAL but indicate that the induction of mutations resulting in enzyme activity decrease efficiently models human FD, consistent with previous studies highlighting the use of zebrafish as a valuable kidney disease model.

Taken together, our data paves the way for a better molecular understanding of kidney phenotypes observed in FD patients and opens new avenues for the identification of novel biomarkers, and the performance of large-scale drug screenings.

## Fund

This work was supported by an open project grant (number HV912233) to Hans-Peter Marti from the Western Norwegian Health Region (10.13039/501100004257Helse vest).

## Submission declaration and verification

This work described has not been published previously, and it is not under consideration for publication elsewhere. This work is approved by all authors and tacitly or explicitly by the responsible authorities where the work was carried out, and that, if accepted, it will not be published elsewhere in the same form, in English or any other language, including electronically without the written consent of the copyright holder.

## Declaration of Competing Interest

Maria Blomqvist has given lectures in symposia and expert meetings sponsored by 10.13039/100004339Sanofi Genzyme, 10.13039/100008373Takeda Pharmaceutical Company and 10.13039/100008484Biomarin Pharmaceutical. The authors declare no conflict of interest. Sabine Leh received speaker fees from Genzyme Sanofi. Einar Svarstad; speaker's fees and travel support from Amicus, 10.13039/100013995Sanofi Genzyme, and Shire; advisory board honoraria from Amicus and 10.13039/100013995Sanofi Genzyme. Camilla Tøndel; consultancy honoraria and/or research support from Amicus, 10.13039/100013995Sanofi Genzyme, Chiesi, Protalix, Idorsia and Freeline.

## Appendix A Supplementary data

Supplementary material Image 1
