# Supplementary material for: Reduced α-galactosidase A activity in zebrafish (Danio rerio) mirrors distinct features of Fabry nephropathy phenotype
Source: Mol Genet Metab Rep. 2022 Feb 17;31:100851. doi: 10.1016/j.ymgmr.2022.100851 (PMC8857658; doi:10.1016/j.ymgmr.2022.100851)

# Supplementary materials

Sup. 1 Guide RNA sequence used for creating the mutant line, and the primer set used for genotyping.

| Item | Sequence |
| --- | --- |
| Guide RNA | 5’-CGTCTTCTAGCAGCAGCAGGGTC**ACC**-3’ |
| Forward primer | 5’-AGAGAACGTAAAGTCGGACTG-3’ |
| Reverse primer | 5’-CCGTTTTCCTTACCCTACGT-3’ |

Sup. 2. Schematic drawing of the mutant generation. In the first step, wildtype fish were crossed, and their eggs were injected with designated CRISPR/Cas9-gRNA complex. The fish were grown, genotyped, and selected heterozygous were back crossed with wildtype fish to refine the mosaic mutations. The produced progeny was genotyped, sequenced, and selected based on the presence of the desired mutation. Later, two heterozygous fish with the same mutation were in-crossed, which produced homozygous mutant fish. This final product fish was used in this study.


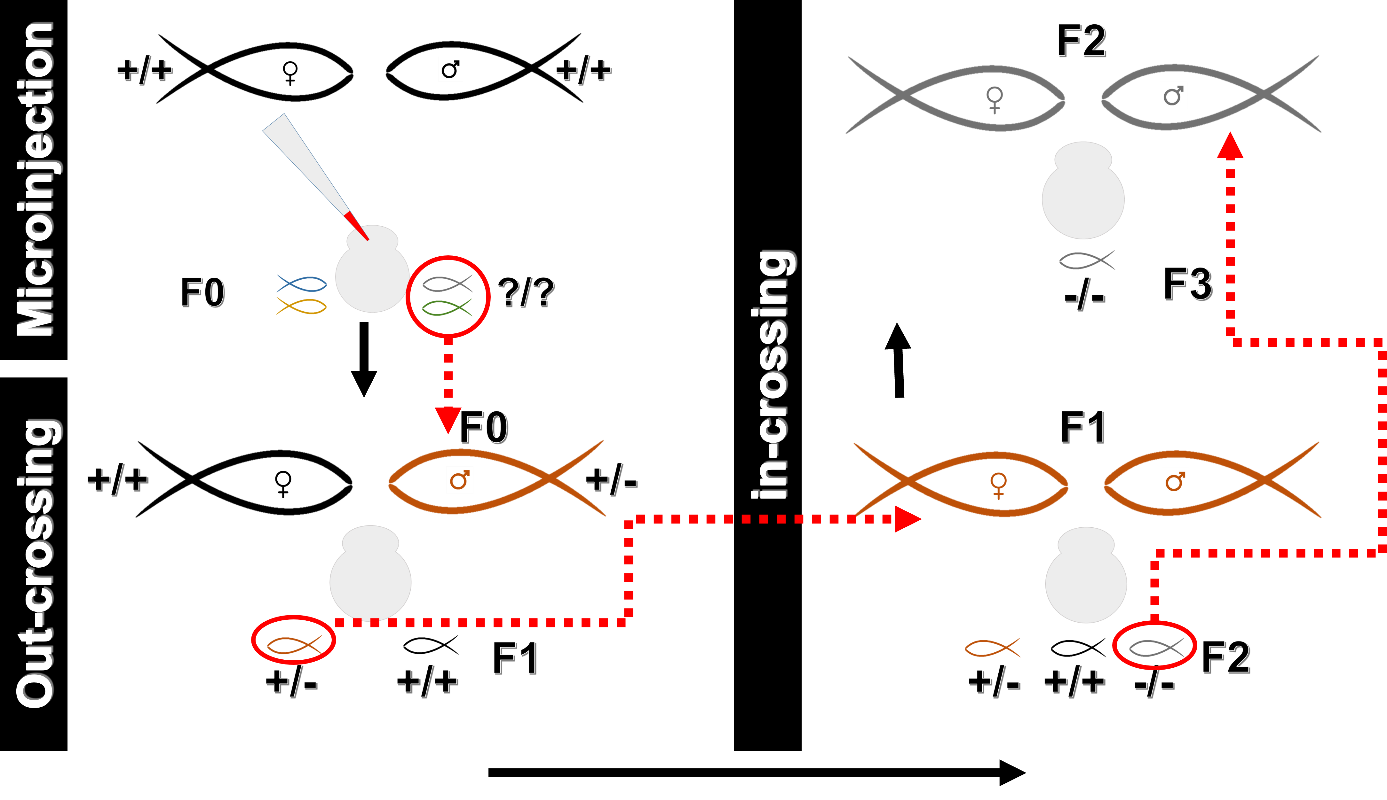


Sup. 3 Chromatographic separation separations (C18 column) of lysoGb3 in kidney tissue lysate. Chromatograms are shown for lysoGb3 with retention time of 2.11 min. for the internal standard (B), no peak was detected in the samples (A, one representative sample, Sample No.=2/genotype).


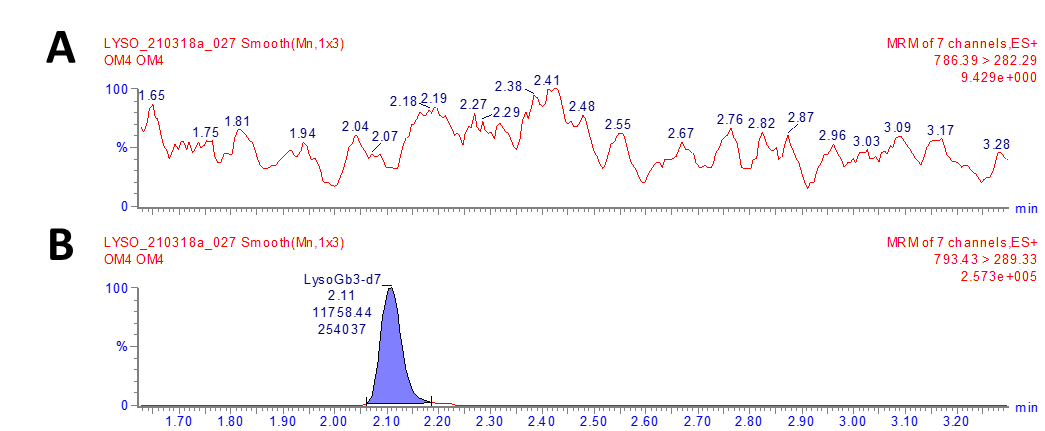


Sup. 4 Validation of the customized alpha-gal antibody on wildtype zebrafish kidney. A: zebrafish kidney where primary antibody is replaced with antibody diluent, B: the same sample and the same region (red circle) where the primary antibody is used. The staining was performed simultaneously. Scale bar (black) = 100µm.


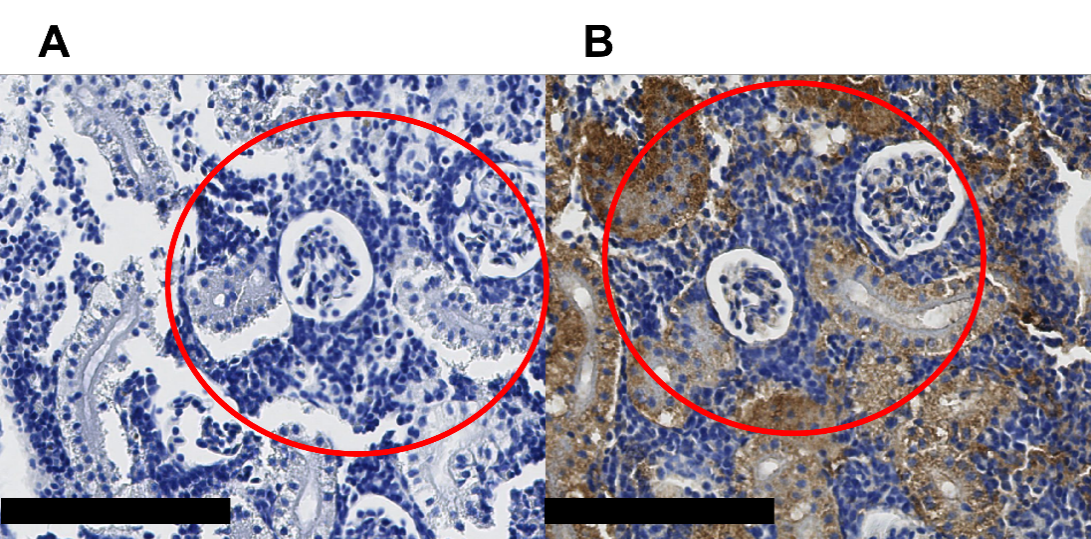


Sup. 5. Genotyping of the mutants. Digestion of the PCR product. L= DNA ladder in base pair bp, C5=PCR product (undigested) control, C5’= PCR product (digested) control. Crossing of mutant^-/-^=100%^-/-^ (samples 1’-8’). Crossing mutant^-/-^ Vs heterozygous^+/-^= 43.75% ^-/-^, 56.25%^+/-^ (samples 9’-24’). Crossing heterozygous^+/-^ = 12.5%^-/-^, 87.5%^+/-^ (samples 25’-32’). Crossing heterozygous parents^+/-^ = 50% +/-, 50%^+/+^ (samples 33’-36’).


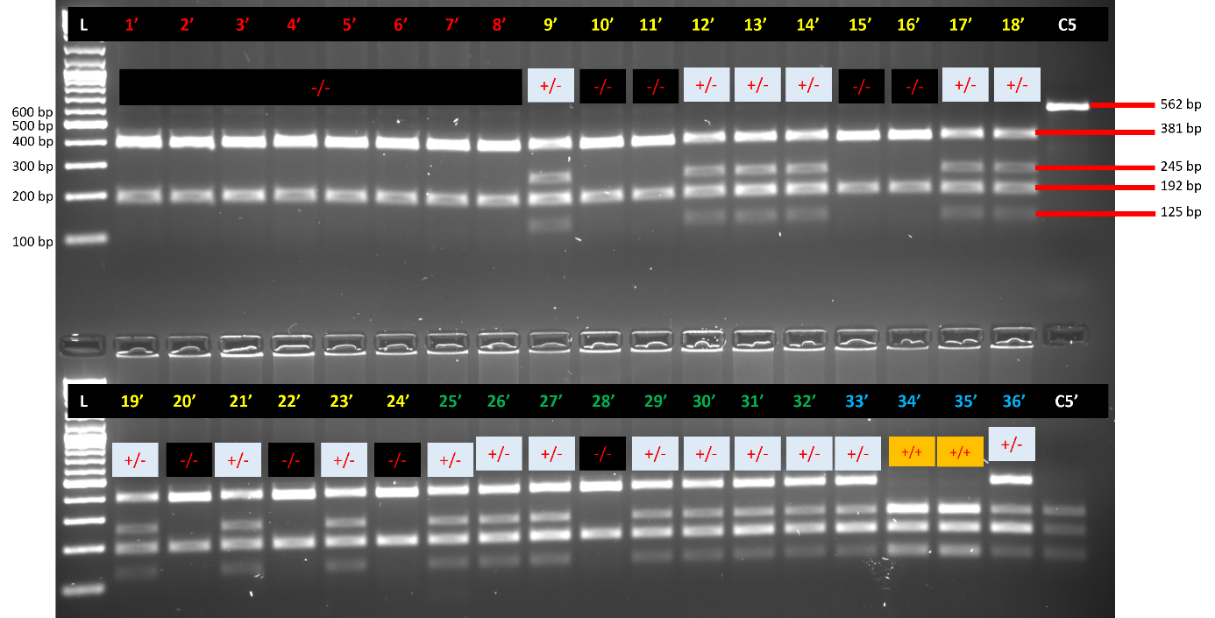


Sup. 6 Mass spectrometric Identification of zebrafish’s proteinuric leaked proteins from three gel bands at 80kDa, 98kDa and 150kDa respectively.

|  | Accession | Description | | MW [kDa] | |  |
| --- | --- | --- | --- | --- | --- | --- |
| Band 80kD | A0A0R4IKS1 | | Transglutaminase 1-like 2 [OS=Danio rerio] | | 96.1 | |
|  | F1R2S5 | | Vitellogenin 5 [OS=Danio rerio] | | 149.3 | |
|  | F1QVX3 | | myosin, heavy chain b [OS=Danio rerio] | | 222.7 | |
|  | A0A0R4IQV3 | | Ankyrin repeat and KH domain-containing 1 [OS=Danio rerio] | | 274 | |
|  | F1RDU8 | | si:dkey-65b12.6 [OS=Danio rerio] | | 304.4 | |
| Band 98kD | X1WH88 | | CUB and zona pellucida-like domains 1, tandem duplicate 2 [OS=Danio rerio] | | 80.9 | |
|  | A0A0R4IKS1 | | Transglutaminase 1-like 2 [OS=Danio rerio] | | 96.1 | |
|  | Q503C7 | | alpha-1,4 glucan phosphorylase [OS=Danio rerio] | | 96.9 | |
|  | A2BIP1 | | ATPase, Ca++-transporting, ubiquitous [OS=Danio rerio] | | 102.6 | |
|  | Q8AX99 | | Actinin alpha 3b [OS=Danio rerio] | | 103.8 | |
|  | Q5U3A4 | | Calcium-transporting ATPase [OS=Danio rerio] | | 110.1 | |
|  | Q6NYQ7 | | Nicotinamide nucleotide transhydrogenase [OS=Danio rerio] | | 112.7 | |
|  | F1R2S5 | | Vitellogenin 5 [OS=Danio rerio] | | 149.3 | |
|  | Q1MTC4 | | vitellogenin 2 [OS=Danio rerio] | | 179.8 | |
| Band 150kD | Q503C7 | | alpha-1,4 glucan phosphorylase [OS=Danio rerio] | | 96.9 | |
|  | A2BIP1 | | ATPase, Ca++-transporting, ubiquitous [OS=Danio rerio] | | 102.6 | |
|  | Q8AX99 | | Actinin alpha 3b [OS=Danio rerio] | | 103.8 | |
|  | Q5U3A4 | | Calcium-transporting ATPase [OS=Danio rerio] | | 110.1 | |
|  | A0JMC2 | | Myhz2 protein [OS=Danio rerio] | | 110.9 | |
|  | F1R2S5 | | Vitellogenin 5 [OS=Danio rerio] | | 149.3 | |
|  | Q1MTC4 | | vitellogenin 2 [OS=Danio rerio] | | 179.8 | |
|  | A0A0R4IRI2 | | si:dkey-65b12.6 [OS=Danio rerio] | | 192.7 | |
|  | F1R6C7 | | myosin, heavy chain a [OS=Danio rerio] | | 222.1 | |

Sup. 7. Proteinuria full gel image not edited. MW=marker in kDa, WT= wild type, Mu1 and Mu2= Mutant sample (2 different samples), C= control (water). White arrow heads pointing to the selected high molecular weight bands which were used for the LC-MS/MS analysis.


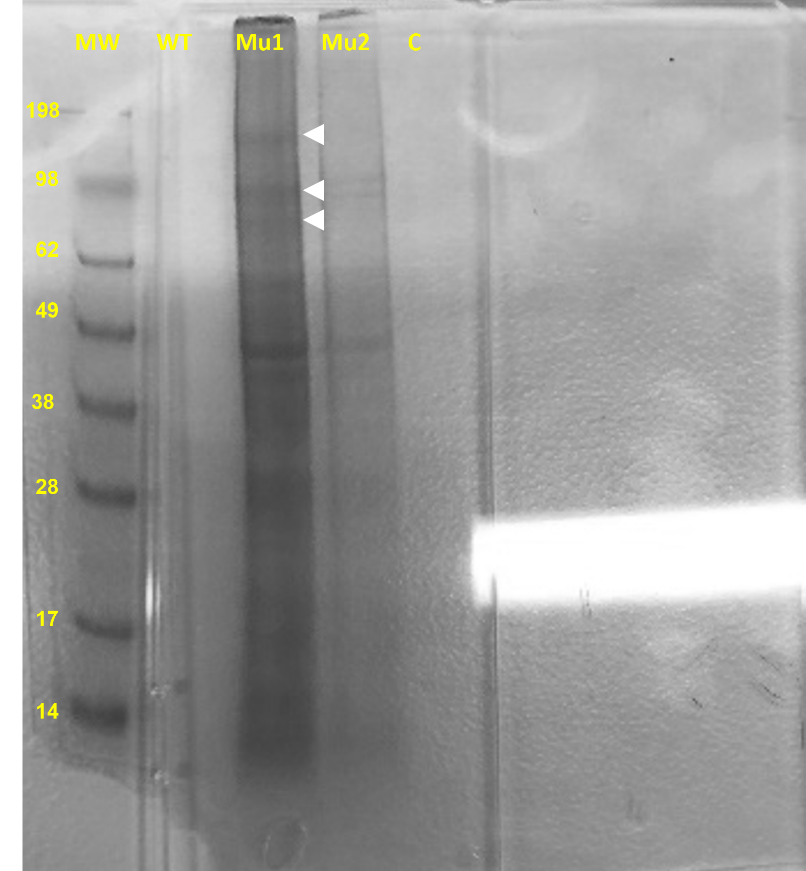


Sup. 8 Observation on the morphology and survival. A: The measurement of size and weight is provided in the figure below, data used to produce this figure is in the table below. YM= young male, YF=young female, OF=old female, OM=old male. Additionally, no spinal curviness is observed. B: Survival during the first 4 day of embryonic development. Total number of embryos per group 150 embryo.


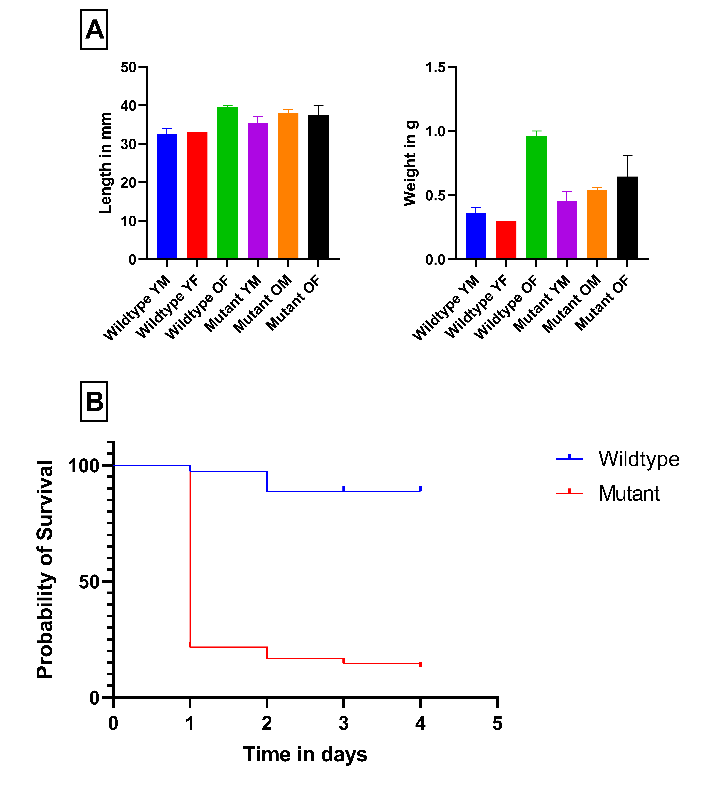


| **#** | **Genotype** | **Gender** | **DOB** | **measure date** | **Age in Months** | **Weight in g** | **Length in mm** | **Spinal curve.** |
| --- | --- | --- | --- | --- | --- | --- | --- | --- |
| 1 | WT | M | 26 March 2021 | 17 October 2021 | 6 | 0.38 | 34 | No |
| 2 | WT | F | 26 March 2021 | 17 October 2021 | 6 | 0.3 | 33 | No |
| 3 | WT | M | 26 March 2021 | 17 October 2021 | 6 | 0.34 | 32 | No |
| 4 | WT | M | 26 March 2021 | 17 October 2021 | 6 | 0.4 | 34 | No |
| 5 | WT | M | 26 March 2021 | 17 October 2021 | 6 | 0.33 | 33 | No |
| 6 | WT | M | 26 March 2021 | 17 October 2021 | 6 | 0.36 | 32 | No |
| 7 | WT | M | 26 March 2021 | 17 October 2021 | 6 | 0.36 | 30 | No |
| 8 | WT | M | 26 March 2021 | 17 October 2021 | 6 | 0.36 | 33 | No |
| 9 | WT | M | 26 March 2021 | 17 October 2021 | 6 | 0.33 | 30 | No |
| 10 | WT | F | 30 August 2020 | 17 October 2021 | 13 | 0.92 | 40 | No |
| 11 | WT | F | 30 August 2020 | 17 October 2021 | 13 | 1 | 39 | No |
| 12 | Mut. | M | 27 March 2021 | 17 October 2021 | 6 | 0.34 | 33 | No |
| 13 | Mut. | M | 27 March 2021 | 17 October 2021 | 6 | 0.39 | 33 | No |
| 14 | Mut. | M | 27 March 2021 | 17 October 2021 | 6 | 0.42 | 35 | No |
| 15 | Mut. | M | 27 March 2021 | 17 October 2021 | 6 | 0.5 | 37 | No |
| 16 | Mut. | M | 27 March 2021 | 17 October 2021 | 6 | 0.49 | 36 | No |
| 17 | Mut. | M | 27 March 2021 | 17 October 2021 | 6 | 0.53 | 37 | No |
| 18 | Mut. | M | 30 August 2020 | 17 October 2021 | 13 | 0.56 | 39 | No |
| 19 | Mut. | F | 30 August 2020 | 17 October 2021 | 13 | 0.48 | 35 | No |
| 20 | Mut. | M | 30 August 2020 | 17 October 2021 | 13 | 0.52 | 37 | No |
| 21 | Mut. | F | 30 August 2020 | 17 October 2021 | 13 | 0.81 | 40 | No |

Sup. 9 Schematic diagram illustrates zebrafish GLA gene with its 7 exons and showing the annotated enzyme important sites and the position of the mutation (exon 5) and the postulated premature stop codon (exon 6). Immunogenic peptides are also shown located at the 7^th^ exon.


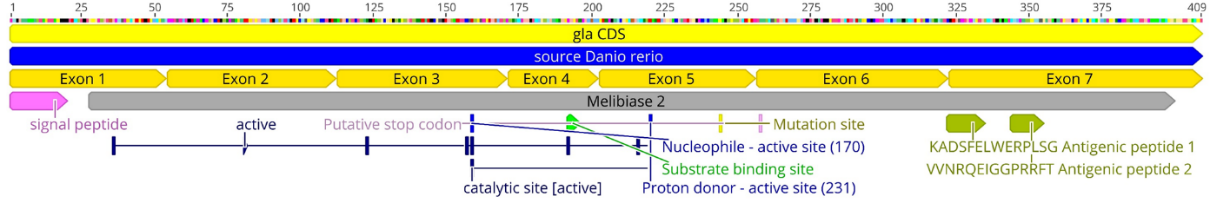

Supplement: Supplementary file 1 — Supplementary material [file mmc1.docx]
